# Severe Form of Bacterial Meningitis After Spine Surgery: A Case Report and Review of the Literature

**DOI:** 10.7759/cureus.13877

**Published:** 2021-03-14

**Authors:** Anuj Gupta, Kuldeep Bansal, Harvinder Singh Chhabra, Pratyush Shahi

**Affiliations:** 1 Orthopaedics and Spine, Triveni Ortho & Spine Center, Delhi, IND; 2 Spine Surgery, Indian Spinal Injuries Center, Delhi, IND; 3 Spine Service, Indian Spinal Injuries Center, Delhi, IND; 4 Orthopaedics, University College of Medical Sciences and Guru Teg Bahadur Hospital, Delhi, IND

**Keywords:** meningitis, spine surgery, complication, dural tear, csf leak

## Abstract

Meningitis after spine surgery is a rare complication. In this report, we aim to discuss the case of a male patient who developed this rare condition after undergoing cervical spine surgery with devastating outcomes. We also engage in a review of the relevant literature.

A 17-year-old boy presented with post-traumatic cervical kyphotic deformity with signs of cord compression. He was operated in three stages, all conducted in a single sitting. There was an incidental cerebrospinal fluid (CSF) leak, which was primarily repaired. On the fourth postoperative day, the patient developed altered sensorium and seizures. Evaluations for clinical signs of meningitis such as neck rigidity and Kernig’s sign were inconclusive. CSF analysis confirmed the diagnosis of meningitis. Thereafter, the patient developed hydrocephalus and intractable infection, for which multiple procedures were done. Finally, we succeeded in controlling the infection, but the patient developed a neurological deficit, which did not resolve even after 2.5 years of follow-up.

The clinical signs and symptoms of meningitis after cervical spine surgery are not very clear or suggestive. A strong index of suspicion should be maintained for the early detection of this condition to prevent devastating complications that result from it.

## Introduction

Postoperative meningitis is a rare complication after spine surgery. It mostly develops after incidental durotomy, which itself is a rare occurrence [1]. The incidence of dural tear varies for different spine procedures; for instance, it is 5.3% for open discectomies and 1.8% for micro discectomies [2]. The rate of incidence increases to 17.4% in revision cases. The most common organisms reported are *Staphylococcus aureus, Escherichia coli, and Enterococcus faecalis* [3]. Due to the low incidence rate of this condition, there is a dearth of data in the literature about the course of the disease and its complications. In this report, we present a case of post-cervical spine surgery with meningitis, which led to a devastating outcome.

## Case presentation

**Figure 1:**
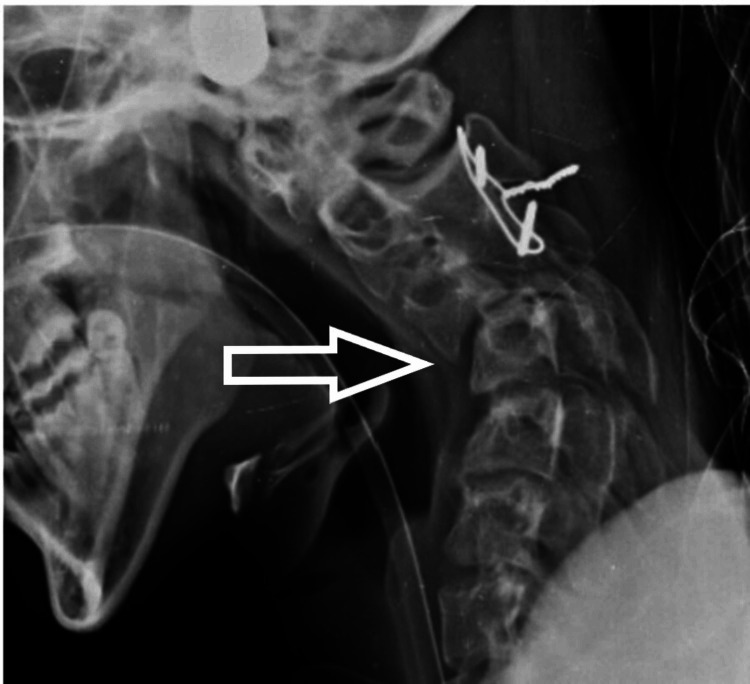
Preoperative X-ray

**Figure 2:**
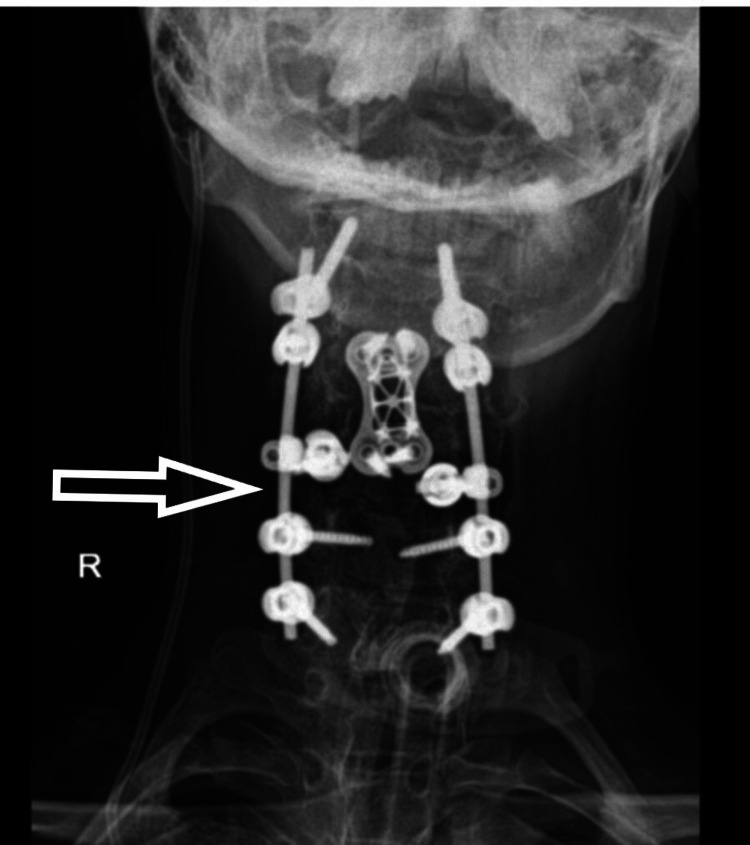
Postoperative X-ray - image 1

**Figure 3:**
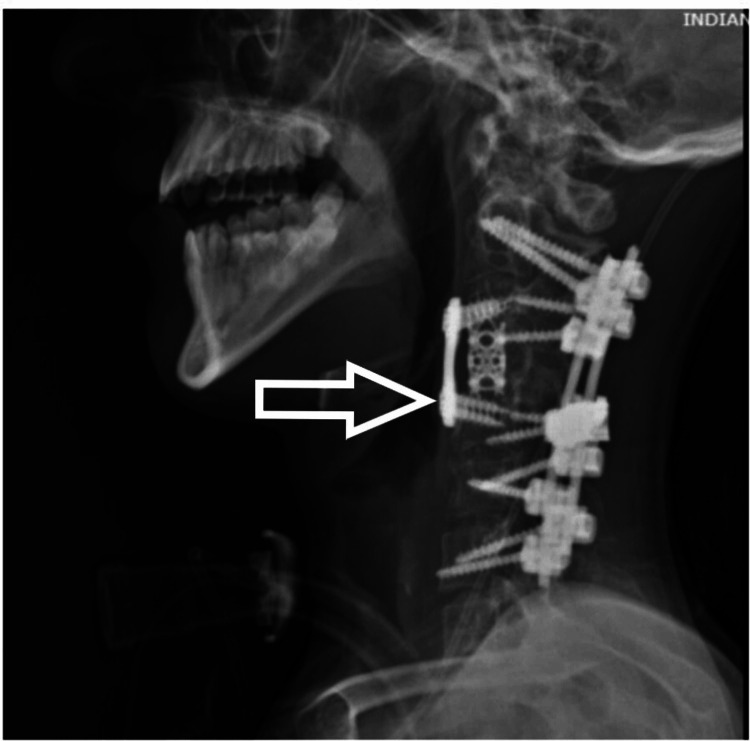
Postoperative X-ray - image 2

A 17-year-old male patient presented to us with weakness in bilateral upper and lower limbs and difficulty in walking for the past six months, which had been insidious in onset and progressive in nature. The patient had a history of a fall two years back and had sustained a neck injury, for which he had undergone C2-3 interspinous wiring elsewhere. On clinical evaluation, his modified Japanese Orthopedic Association (mJOA) score was 11/17 with Nurick Grade 3. The patient had a Medical Research Council (MRC) Scale score of 4/5 in all four limbs. On sensory examination, he had an abnormal touch and pinprick sensation below C6 dermatomes. All deep tendon reflexes (DTR) were exaggerated with the Babinski sign present. On imaging (Figure 1), the patient had cervical kyphotic deformity below the site of interspinous wiring with signal intensity changes in the cord on T2WI MRI. The patient was taken up to the OT, and a three-stage procedure was performed in a single sitting. In the first stage, the anterior corpectomy of C4 was done. In the second stage, the patient was flipped and a posterior stabilization C2-7 with decompression with deformity correction was done. Finally, the patient was flipped again, and the mesh cage and plating were done anteriorly (Figures 2, 3).

**Figure 4:**
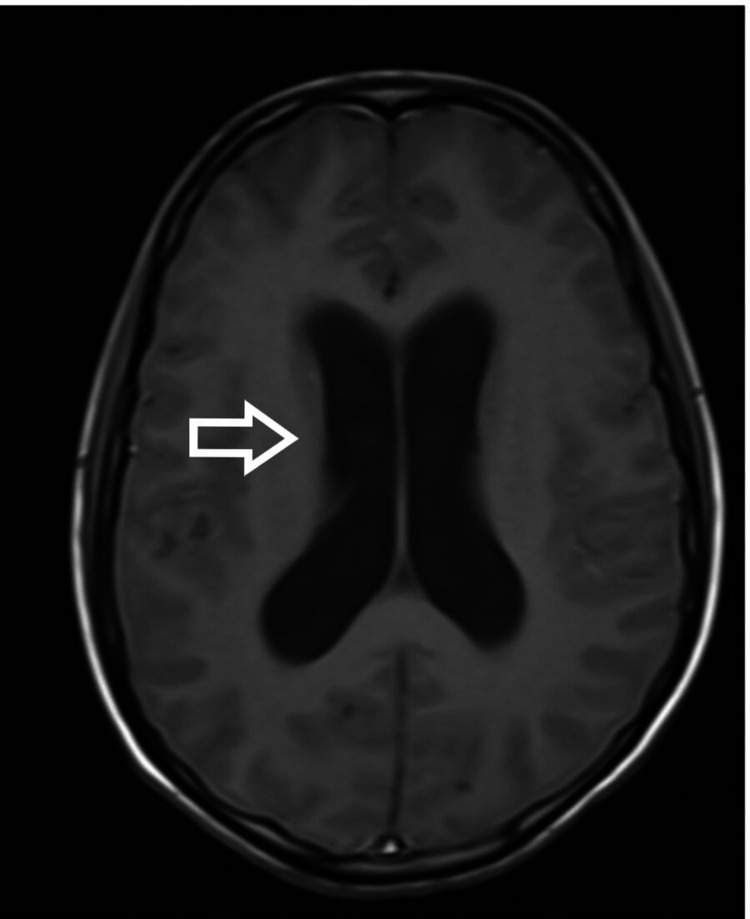
MRI brain showing evidence of hydrocephalus with leptomeningeal enhancement on the post-contrast image - view 1 MRI: magnetic resonance imaging

**Figure 5:**
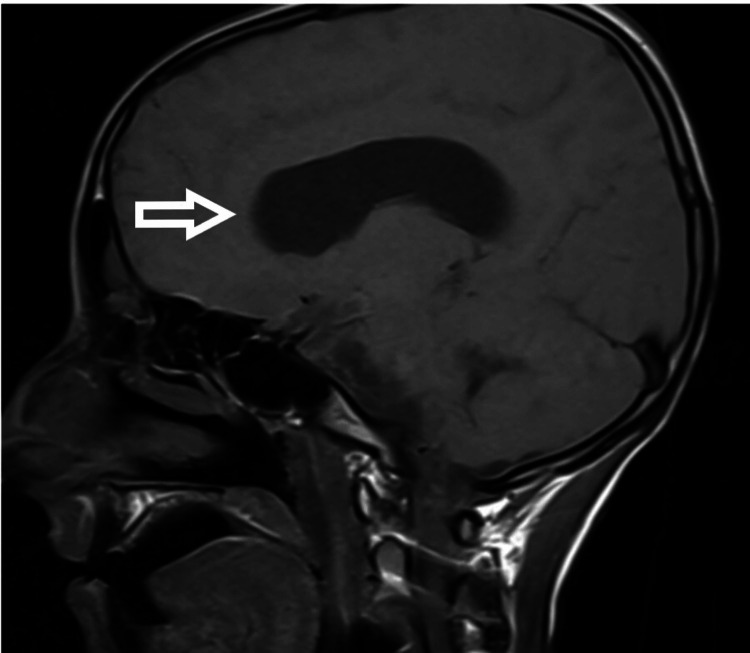
MRI brain showing evidence of hydrocephalus with leptomeningeal enhancement on the post-contrast image - view 2 MRI: magnetic resonance imaging

**Figure 6:**
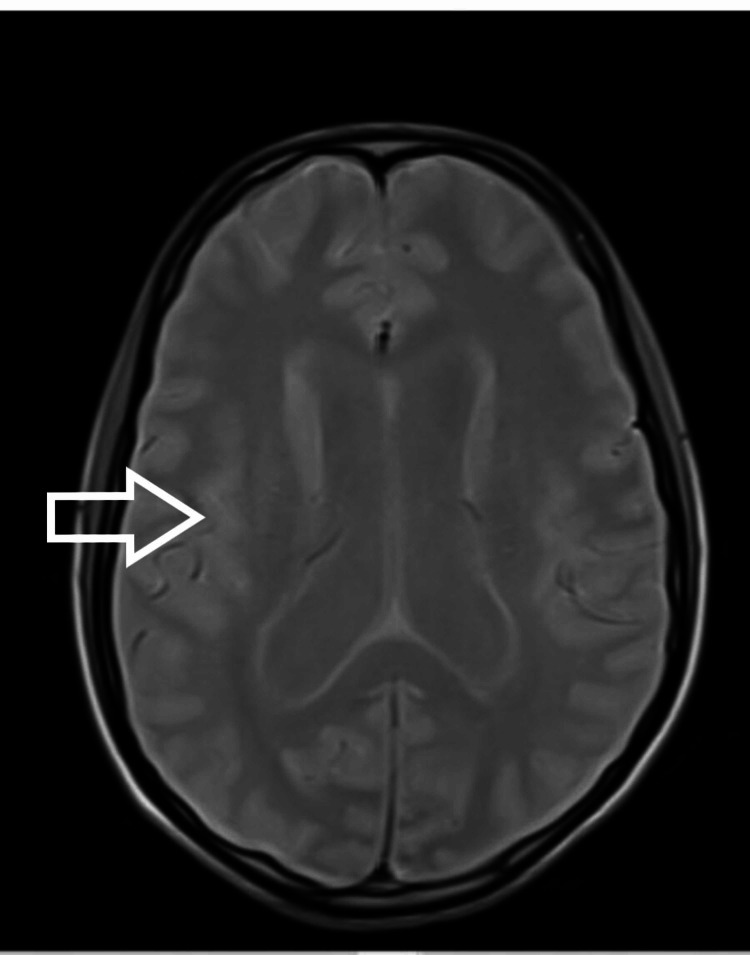
MRI brain showing evidence of hydrocephalus (A, B) with leptomeningeal enhancement on the post-contrast image (C) - view 3 MRI: magnetic resonance imaging

Intraoperatively, there was cerebrospinal fluid (CSF) leakage due to a dural tear on anterior surgery, which was repaired. A lumbar drain was also inserted (we normally remove the lumbar drain after 48 hours). Immediate postoperative neurology was the same as preop and the patient was extubated. On day four postoperatively, the patient had difficulty in breathing and altered sensorium without any focal neurological deficit [Glasgow Coma Scale (GCS): 14/15]. The patient was again intubated. The surgical wound was without erythema, swelling, or discharge. Neck rigidity, Kernig's sign, and Brudzinski's sign could not be assessed as the patient was sedated. Initial blood parameters showed high total leukocyte counts with a predominant increase in neutrophils. CSF sample from lumbar drain showed pus cells and Gram-negative coccobacilli with raised protein and low glucose. After consultation with the Infectious Diseases department and the Department of Neurology, empirical antibiotic treatment with vancomycin (1g q12h iv) and meropenem (2g q12h iv) was started. After proper culture and sensitivity reports, Klebsiella was found and antibiotics were changed to inj colistin, inj cotrimoxazole, and inj meropenem. The patient was tracheostomized in light of poor respiratory effort and the need for prolonged ventilatory support. In the following days, the patient developed a loss of consciousness and seizure. MRI brain (Figures 4, 5, 6) showed dilated ventricular system with periventricular edema and multiple loculated collections in the brain stem and cortex.

**Figure 7:**
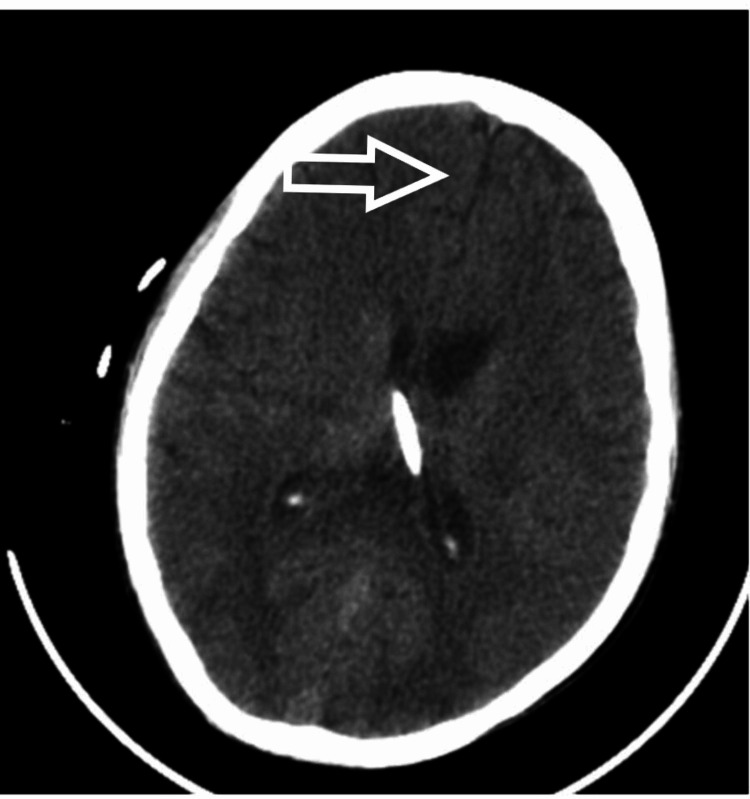
NCCT head showing hydrocephalus and midline shift - view 1 NCCT: noncontrast computed tomography

**Figure 8:**
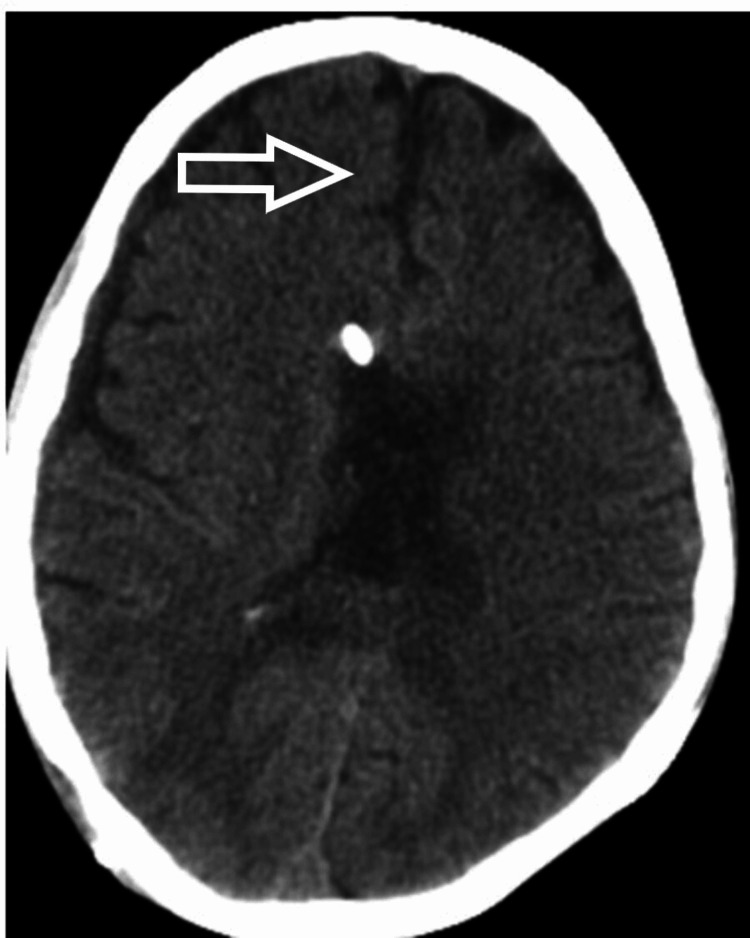
NCCT head showing hydrocephalus and midline shift - view 2 NCCT: noncontrast computed tomography

**Figure 9:**
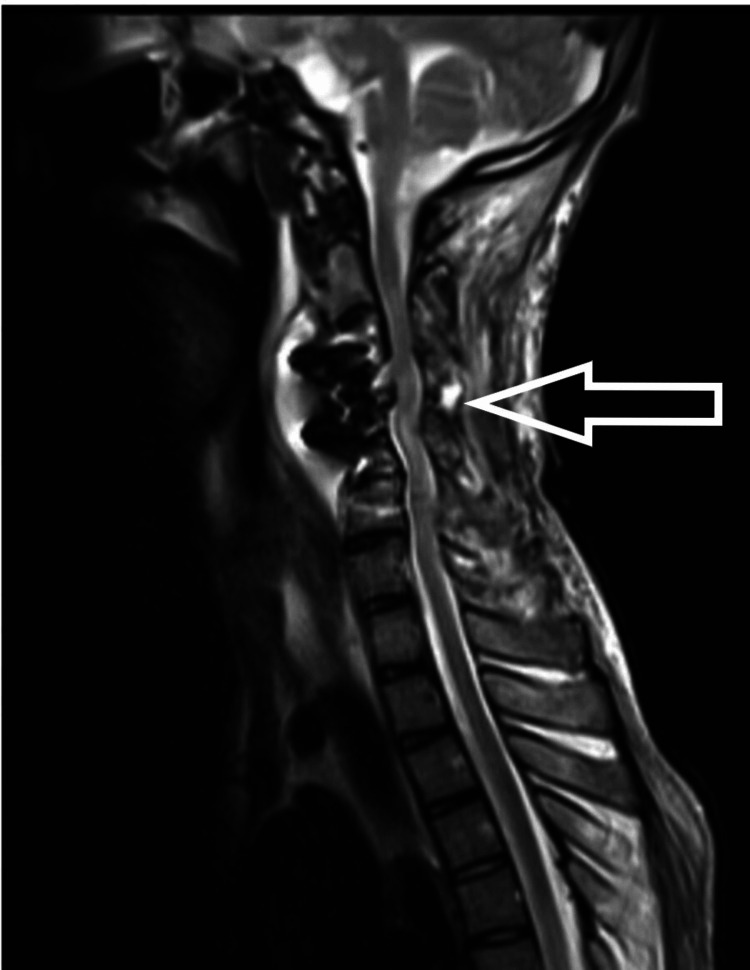
MRI showing diffuse leptomeningeal enhancement with pre-paravertebral collection (15 days after surgery) MRI: magnetic resonance imaging

The patient was taken to the OT for the placement of frontal Ommaya reservoir with intraventricular tigecycline continued through the reservoir. He also underwent an endoscopic third ventriculostomy. There was persistent hydrocephalus despite the placement of the reservoir. After the settlement of the infection, a biventricular ventriculoperitoneal (VP) shunt with a Y connector was applied. Due to the deterioration in the neurology, a noncontrast CT (NCCT) of the brain (Figures 7, 8) was done, which revealed a small bleed in the left temporal lobe, and hence the repositioning of the shunt was done. MRI of the cervical spine (Figure 9) was also done, which showed adequate decompression and proper positioning of implants and no further extension of cord signal intensity changes. After a prolonged antibiotic course and multiple procedures, we succeeded in controlling the infection, but a neurological deficit of the patient did not resolve. With an extensive and comprehensive rehabilitation program, the patient was weaned off from the ventilator. After 2.5 years of surgery, the patient is breathing spontaneously with grade 1 power in the C8 myotome and grade 0 power in the rest of the myotome.

## Discussion

Postoperative bacterial meningitis is a rare complication following spine surgery, but it can sometimes lead to devastating outcomes with long-term sequelae. The overall incidence of dural tear ranges from 0.3% to 13% depending on the type and site of surgery [4]. However, in cervical spine surgeries, the incidence has been reported to be 1% [5]. Due to the rarity of meningitis as a complication after dural tear, the prevalence rate of this complication has not been reported in many studies. The available studies report the incidence to be around 0.18% [6].

The most common cause of meningitis is bacterial infection [3]. The clinical symptoms of meningitis include the triad of fever, neck stiffness, and altered sensorium [7]. The presence of all three features is rare in cases of meningitis after spine surgery and especially after cervical spine surgery. Our patient presented with initial symptoms of altered sensorium and poor respiratory effort. The atypical presentation in our patient could be attributed to the fact that postoperatively, as per protocol, we start analgesics to counter pain sensation, which suppresses the inflammatory response and the fever does not manifest. Neck rigidity is difficult to comment upon in patients who underwent cervical spine surgery with instrumented fusion. Also, headache, neurological deficits, and seizures have been reported as symptoms of meningitis with less frequency [6]. Our patient developed seizures and neurological deficits after a few days during the course of the hospital stay.

A lumbar puncture should be performed for every patient with suspected meningitis if there are no contraindications. Blood cultures are also suggested for those who develop a fever. Brain CT is recommended to rule out other causes of consciousness disturbance; however, imaging studies should not delay antibiotic treatment. In addition, electrolyte balance and fluid management with the use of intravenous hydration have been shown to be beneficial for patients with meningitis [8]. We did a lumbar puncture on the fourth postoperative day when we had the first suspicion of meningitis. MRI of the brain with contrast was also performed, which showed dilated ventricular system with periventricular edema with multiple loculated collections in the brain stem and cortex. In contrast MRI, hyperintensities were noticed at the frontoparietal subcortical white mater, lentiform nucleus, thalamus, and internal capsule. NCCT of the head was consistent with the findings of MRI and showed a shift of midline to the left side in addition to hemorrhage in ventricles, thalamus, and brainstem. CSF analysis was suggestive of pus cells with all findings supportive of bacterial meningitis. A blood picture can be misleading as there is leukocytosis after every surgery. Hence, the trend of leucocytosis must be noted.

Empirical antibiotics with good blood-brain barrier penetration should immediately follow the lumbar puncture. The antibiotics should cover common pathogens and should achieve adequate concentration in CSF [9]. Antibiotics can be modified according to the available sensitivity reports, which normally takes 72 hours. This delay should be avoided to prevent adverse outcomes such as mortality and unrecoverable neurological deficits [10]. We started inj vancomycin and inj meropenem, which cover common organisms and also have good penetration in CSF. Though many studies have reported good outcomes with conservative management in postoperative meningitis, we did not observe any improvement with intravenous antibiotics in this patient. Hence, we planned for the placement of the Ommaya reservoir for the direct instillation of antibiotics.

After several efforts and multiple procedures in our patient, we succeeded in controlling the infection; however, he did not improve neurologically even after 2.5 years of follow-up. To the best of our knowledge, our is the first article to report on such a devastating outcome after the development of meningitis following cervical spine surgery. There had been many published articles on the development of meningitis after incidental durotomy in lumbar spine surgeries [1,6,9,11-13], but there is a dearth of data in the literature regarding the same after cervical spine surgery. Table 1 provides a summary of the previous articles in the literature that mention meningitis after spine surgery.

**Table 1:** A summary of previous studies in the literature mentioning meningitis after spine surgery VP: ventriculoperitoneal; TLIF: transforaminal lumbar interbody fusion

Author	Sample size	Treatment	Spinal procedure	Complication	Intraoperative durotomy
Lin et al., 2014 [6]	21 patients	Conservative intravenous antibiotics. Three patients required surgery for dural repair	Degenerative spondylolisthesis in seven patients, degenerative lumbar scoliosis in seven patients, lumbar spinal stenosis in one patient, herniated intervertebral disc in four patients, and segmental instability in two patients	None	10 out of 21 had incidental durotomy
Zhang et al., 2017 [9]	One patient	Conservative followed by dural repair and debridement twice	L4-L5 TLIF for lumbar canal stenosis	None	Incidental durotomy
Chaichana et al., 2007 [11]	One patient	Conservative followed by neurosurgical intervention in the form of VP shunt	Lumbosacral spine surgery	Aphasia due to cerebral vasospasm	Durotomy
Todd et al., 2008 [12]	One patient	Conservative	L4-5 discectomy	Aseptic meningitis	No durotomy
deFreitas and McCabe, 2004 [1]	One patient	Dural repair surgical + IV antibiotics	L4-5 discectomy	Persistent headache	Dural tear but no arachnoid breach
Twyman et al., 1996 [3]	Four patients		Lumbosacral fixation, lumbar decompression, cervical decompression, L4-5 discectomy	No mortality	One of four had incidental durotomy
da Costa et al., 2007 [13]	One patient	Surgical treatment	Scoliosis surgery	Persistent back pain (delayed presentation)	No durotomy

## Conclusions

Meningitis after spine surgeries is a rare complication; however, clinicians should maintain a strong index of suspicion so that early and prompt treatment could be initiated to prevent long-term complications. Also, the signs and symptoms related to meningitis after cervical spine surgery are not clear or suggestive, which is another reason for the delayed diagnosis of the condition.
